# Living with Lions: The Economics of Coexistence in the Gir Forests, India

**DOI:** 10.1371/journal.pone.0049457

**Published:** 2013-01-16

**Authors:** Kausik Banerjee, Yadvendradev V. Jhala, Kartikeya S. Chauhan, Chittranjan V. Dave

**Affiliations:** Wildlife Institute of India, Chandrabani, Dehra Dun, Uttarakhand, India; Australian Wildlife Conservancy, Australia

## Abstract

Rarely human communities coexist in harmony with large predators. Most often communities suffer due to predation on their stock while large carnivores suffer losses and at times extirpation due to retaliation. We examine the mechanisms permitting the coexistence of Asiatic lions (*Panthera leo persica*) and pastoral communities (*Maldhari*s) in the Gir forests, India. We monitored six *Maldhari* settlements between 2005 and 2007 to quantify seasonal livestock holding, density and losses due to predation and other causes. Lion density, estimated by mark recapture, was 15±0.1 SE/100 km^2^. Livestock density, estimated by total counts, ranged between 25/km^2^–31/km^2^ with buffaloes being most abundant. Average livestock holding of *Maldhari* families was 33±3 SE. Lions predated mostly on unproductive cattle (30%). Scat analysis (n = 165), predation events (n = 180) and seven continuous monitoring sessions of 1,798 hours on four radio-collared lions estimated livestock to contribute between 25 to 42% of lions’ biomass consumptions, of which only 16% was predated; rest scavenged. With free grazing rights within Gir forests, *Maldhari*s offset 58±0.2 SE% of annual livestock rearing cost in comparison to non-forest dwelling pastoralists. With government compensation scheme for livestock predation, this profit margin augmented to 76±0.05 SE%. Lion density was higher in areas with *Maldhari* livestock in comparison to areas without livestock. Thus, the current lifestyles and livestock holdings of *Maldhari*s seem to be beneficial to both lions and local pastoralists. We conclude that a combination of strict protection regime for lions, *Maldhari*s’ traditional reverence towards lions and the livelihood economics permit the delicate balance of lion-*Maldhari* coexistence. Indefinite increase in human and livestock population within Gir might upset this equilibrium undermining the conservation objectives. We see no end to compensation programs worldwide as they constitute a crucial element needed for human-carnivore coexistence.

## Introduction

Rarely do forest-dwelling pastoral communities coexist in harmony with large predators. Either the communities suffer substantial economic loss due to predation on their stock and/or large carnivores suffer heavy losses and even extirpation due to retaliation [1], [2]. Understanding people-carnivore relationship, therefore, becomes crucial especially for the conservation of large carnivores [3], [4]. Although large carnivores sometimes kill humans [5], [6], the major form of conflict arises due to their habit of predating livestock and the resulting threat on economic security of the pastorals [4]. Human communities react differently to this conflict depending on their religious beliefs, customs, cultures, actual and perceived magnitudes of economic losses and the legal status of carnivores [7]. Reactions range from total extermination of large carnivores [8], occasional removal of problem animals [9], [10] to tolerance and coexistence [11].

In a country like India which is home to approximately 1.2 billion people [12], the majority (70%) being rural; forest resources have been part of traditional livelihoods for generations [13]. India’s pre-independence (1947) colonial exploitative forest policies and subsequently post-independence exclusionary forest management often gave rise to polarized conservation debates about the rights of forest-dwelling communities [14], [15]. Politics of ecology becomes more contentious with the pro-people groups often arguing about the merit of conservation governances that alienates traditional forest-dwellers’ access to forests and their resources, while the livelihood economics of forest dwellers are marginalized due to wildlife damage and poor access to markets [16]. The contrary view by preservationists is that consumptive use by an increasing population of forest dwelling communities is unsustainable and detrimental to biodiversity conservation [17], [18].

Two-thirds of India’s wildlife reserves are grazed by livestock [19] where they are often predated upon by large carnivores [20]. Traditional cultural, ethical and religious reverence towards life forms combined with recent legal protection is important in contributing to the continued survival of large carnivores in India [21], [22], [23], [24]. Due to the changing values of a global economic world it is likely that even in rural areas these values will ultimately determine the fate of large carnivores [25]. To date pastoralist communities have shown tolerance to the presence of lions in the Gir forests. Our objective was to assess whether this tolerance was supported by economics.

At the onset of the nineteenth century, Asiatic lions (*Panthera leo persica*) became restricted to the Gir forests of western India and their numbers declined to around 50 individuals due to hunting and habitat loss [26], [27]. Owing to the timely and stringent protection by the Rulers of Junagadh and subsequently during the post-independence by the State-run forest department; Gir lions have increased to about 400 and dispersed into a large tract of agro-pastoral landscape adjoining the Gir forests [28], [29].

The Gir Forests have been inhabited by semi-nomadic pastoral communities called *Maldhari*s for the past one and a half century [30]. Their religion is Hinduism and they have strong ethics and sentiments towards nature and natural resources [11]. They are primarily vegetarian and keep livestock for sale of dairy products. Due to their long history of living with lions that often predate on their livestock, it would be important to understand the underlying mechanisms that permit coexistence. In this article we quantify predation losses of livestock, estimate lion densities and diet and evaluate the economics of rearing livestock in lion habitats. We examined the notion that the tolerance of the *Maldhari*s towards lions [11], [31] is not solely due to their beliefs and cultural sentiments but also because it is economically more profitable to live with lions.

## Methods

### Ethics Statement

All permissions to carry out the field research were obtained from the Office of the Chief Wildlife Warden, Gujarat State and Ministry of Environment and Forests, Government of India under the provisions of the Wildlife (Protection) Act, 1972, Government of India. Livestock counts were conducted with permission from their owners without any coersion.

### Study Area

**Figure 1:**
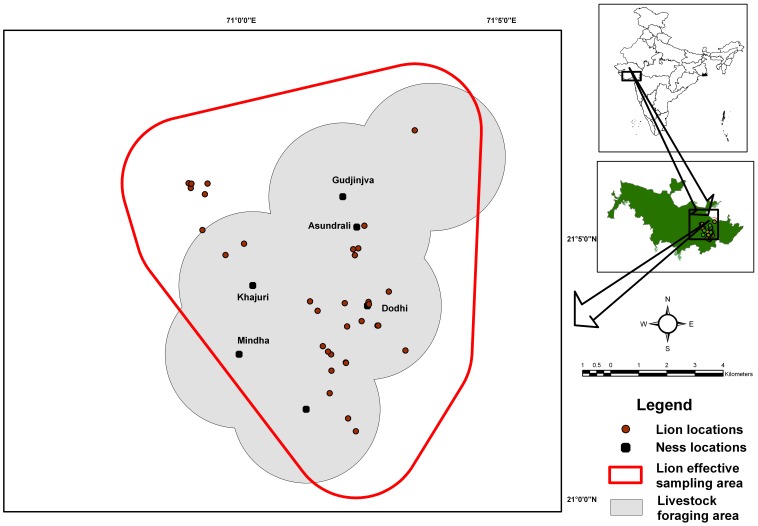
Study site within the Gir forests showing locations of different study *Ness*es buffered by average livestock foraging area, lion capture points and effective lion trapping area. The maps inset show the location of the Gir PA in India and the study site within the eastern part of the Gir forests.

Gir Protected Area (PA) [1,883 km^2^, 20°57′ to 21°20′ N latitude and 70°27′ to 71°13′ E longitude] is a dry deciduous forest [32] situated in Gujarat province, western India (Fig. 1) and is made up of a Sanctuary (with human settlements and regulated grazing and other rights; [24]) covering 1,153 km^2^, a 259 km^2^ National Park (devoid of humans) and 471 km^2^ of additional reserve, protected and unclassified forests. Gir PA has a semi-arid climate with an average minimum and maximum temperature ranging from 5° to 38°C and an average rainfall of 980 mm [31]. Rugged hilly terrains form the catchments of seven perennial rivers. Dominant vegetation included *Tectona grandis*, *Anogeissus* spp, *Acacia* spp and *Ziziphus* spp.

Gir has a diverse assemblage of wild fauna. Apart from the last free-ranging population of the Asiatic lion, some of the other carnivores are leopard (*Panthera pardus*), striped hyena (*Hyaena hyaena*), jackal (*Canis aureus*) and ratel (*Mellivora capensis*). Major wild prey species of lions were chital (*Axis axis*), sambar (*Rusa unicolor*), nilgai (*Boselaphus tragocamelus*) and wild pig (*Sus scrofa*) [31].

Gir Protected Area has 50 *Maldhari* settlements (*ness*es). A *ness* consists of a cluster of thatch and mud hutments of 3–20 *Maldhari* families. [11], [31], [33]. Each *Maldhari* family rears about 20–100 regionally famous indigenous breed of livestock, primarily Jafrabadi breed of buffalo (*Bubalus bubalis*) and Gir breed of cattle (*Bos indicus*). Often one or two camels (*Camelus dromidarius*) are kept for carrying fuel wood and fodder. The sale of dairy products has always been the mainstay of *Maldhari*s’ traditional economy [33]. Our study area covered the livestock grazing areas of a cluster of six *ness*es namely Asundrali, Dodhi, Gudjinjva, Khajuri, Leriya and Mindha (Fig. 1) which represent a typical scenario across Gir PA.

### Lion Density Estimation

We estimated lion population using closed-population mark-recapture [34]. We used cues, including tracks, roars and alert behavior of prey to locate lions. The entire study area of eastern Gir PA was systematically searched by vehicle and on foot within a period of 3–4 days which represented a single occasion. A total effort of 53 days representing 17 occasions was expended. We approached lions within 10–30 meters to determine their whisker spot patterns with binoculars, and by a 15 to 60 X spotting scope. We individually identified lions (>1.5 year) from their unique whisker spot patterns and other permanent unique marks [35]. Close-up color photographs using an 80–400 mm zoom lens were taken of both sides of the face and a full-face view to supplement field drawings [36], [37]. Capture histories of individual lions were used to make an X matrix [34], formally tested for population closure [38] and analyzed using program CAPTURE [39] to deduce population size. The effectively sampled area was estimated by creating a polygon joining the outermost lion locations buffered by a width estimated by half of the mean maximum distance moved (½ MMDM) by recaptured lions [40], [41].

### Livestock Population and Density

A total head count of livestock in each *ness* was carried out. Livestock were counted during evening hours when all livestock were corralled for the night. We recorded data on number and demographic structure of the livestock belonging to each family in a *ness*. We classified livestock as calf, juvenile, sub-adult and adult of both sexes. Adult female livestock were further classified into a) milk yielding, b) temporary dry but breeding age and c) non-productive. Seasonal livestock grazing circuits were estimated and mapped by accompanying three livestock herds from each *ness* in each season from early morning, when they leave to forage in the forest, till they return to the *ness* and were corralled for the night. Data was recorded on distance moved and linear displacement of livestock herds from the *ness* sites from 50 grazing circuits in the form of GPS (Garmin International, Kansas, USA) track logs [42]. Age-gender-productivity class composition of grazing herds as well as their spatial arrangement in a herd was also recorded at every 500 meter interval. Each *ness* site was buffered with its average seasonal foraging radius to compute the foraging area in a GIS map using program Arc GIS (ESRI, Redlands, CA). We calculated seasonal livestock density as the total number of livestock divided by the total foraging area [42].

### Lion Food Habits

Lions’ diet was determined by analysis of 165 lion scats [43], [44] and by monitoring of four radio-collared lions continuously for 5–12 day sessions (detailed below) within the study area. Lion scats were distinguished from those of other predators, particularly leopard scats, based on associated signs, tracks and size [45]. We did not include ambiguous scats in the analysis. Prey remains such as hair, bones, hooves, quills and teeth of the prey consumed were identified to species using reference samples [46], [47]. Data were analyzed as frequency of occurrence and percent occurrence. We assessed adequacy of sample size by plotting the cumulative proportional frequency of occurrence against number of analyzed scat samples of each prey item [48]. We used 1,000 bootstrap iterations [49] using SIMSTAT [50] to generate 95% confidence intervals on frequency of occurrence of different prey items in the lions’ diet.

Due to a differential surface area to volume ratio of small versus large prey, the frequency of occurrence data was corrected to arrive at biomass consumption per collectible scat [51], [52]. We used Ackerman’s equation [developed for cougar (*Felis concolor*)] to convert frequency of occurrence into biomass assuming lions to have a similar digestive physiology as cougars. The equation was *y = *1.980+0.035 *x*, where *y* is the biomass of prey consumed (kg) to produce a single field collectable scat and *x* is the average body weight of the prey species (kg). The body weights of the potential prey species were taken from literature [53], [54]. Prey densities [42] were used as availability. We compared counts of each prey item in the scats with the estimated prey availability using 1,000 bootstrap iterations in program SCATMAN [55], [56] to assess selectivity [57] in utilization. Observed and expected proportions of prey species in the scats were then compared using a G test [58] with two tailed α* = *0.05 level. If there was a pattern of overall selective prey utilization, lions’ use of each prey species as calculated by the program SCATMAN was further inspected. Food preference of lions in the study area was also computed by Jacob’s Index [59] due to its lower bias, smaller confidence intervals with low heterogeneity and freedom from non-linearity compared with other electivity indices [60].

Although frequency of occurrence in scats is a reliable technique for understanding the range of diet items, the method usually cannot distinguish between prey that are killed or scavenged [61], [62]. Consequently occurrence of livestock in the lions’ diet is unreliable to assess lion-*Maldhari* conflict. Therefore, we additionally followed four radio-collared lions on foot and/or four-wheel drive for seven sessions ranging from continuous 192 hours to 360 hours per session to understand the starve-feed cycle of lion foraging behaviour and distinguish between predation and scavenging events [63], [64]. A total of 1,798 hours of monitoring data was recorded during the study period. During this duration, lions were kept in view or within 100 meter from the observers day and night. Lions in Gir are regularly exposed to humans on foot; we further habituated each radio-collared lion for 1–3 days by following it on foot prior to data collection. Radio-collared lions were tolerant to our presence within 20 m without any obvious alteration in their behavior. During dark nights, a flashlight was used at intervals of 30–60 minutes to ascertain lion location apart from the radio signals. All predation and the scavenging events by the lions were recorded during continuous monitoring. Feeding interval was defined as the time lapse between two subsequent feeding events.

On average 75% of the biomass of each carcass/kill greater than 40 kg was observed to be utilized by the predators [65]. We estimated livestocks’ contribution to lions’ diet from lion numbers in the study area obtained from lion density multiplied by daily intake requirement (7.3 kg/day/lion, [45]), scat analysis and continuous monitoring of radio-collared lions in the study area.

### Livestock Depredation Pattern

At each study *ness* a local *Maldhari* was employed to provide information to the authors in the event of a livestock death. KB and/or KSC visited the *ness* site of the mortality event within 24 hrs and recorded data on the time of day of each attack, the number, species and age-sex-productivity class of livestock killed, approximate weight of the predated individual, name of the owner and the identity of the predator. Livestock that died due to natural causes were generally dumped at specific sites outside the *ness*es. We recorded scavenging events by large carnivores which were identified based on direct sightings, vocalization and signs. Information from the owners of dead/predated livestock was obtained on the market price of the livestock and if they had claimed compensation from the Government under the current livestock depredation scheme. The compensation claims were cross validated from the Forest Department’s records.

The monetary value of livestock was assigned in accordance with average prevalent market rate (Table S1). We compared this with the present compensation scheme provided by the Gujarat State Forest Department (Table S1) and the proportion of predation events claimed for compensation from the Government to estimate the offset of the capital loss incurred by the *Maldhari*s due to livestock predation.

### Lion Carrying Capacity

In order to understand the relative significance of wild ungulates and *Maldhari* livestock in maintaining lion density in the study area, we used a regression model [66] that related prey biomass and lion density to estimate the ecological carrying capacity of the eastern Gir for lions. There are several approaches to indirectly predict carnivore density at a site; but studies have shown that it can be obtained more reliably by regressing against prey biomass [67]. The carnivore density derived from this relationship only works as long as no other mechanisms besides prey availability limit a carnivore population. We used prey biomass for predicting lion carrying capacity in our study area as other major top-down limiting factors like trophy hunting and incidence of epizootics [68], [69] were not prevalent in Gir [70]. The model [66] based on lions’ preferred prey species was used. The equation was y = −2.158+0.377×(r^2^ = 0.71, n = 23) where *y* is the log_10_ of lion density and *x* is the log_10_ of preferred prey biomass [66]. We deduced prey biomass of different species by multiplying their densities [42], [71] with their respective unit weights. Since all the livestock units were not available for lion predation, we therefore assessed the lion carrying capacity for three different scenarios; i) no livestock biomass (depicting a situation where there were no *Maldhari* livestock inside the Gir forest), ii) 100% livestock biomass available and iii) 24% (based on our data of feeding events and predation we considered all carcasses of dead livestock and a proportion of dry females, sub-adults and calves that foraged within the forest to be available to lions; this proportion was about 24% of the total livestock population). This enabled us to examine the relative importance of different levels of livestock biomass in sustaining lion population in our study site.

### Cost of Lion Predation on *Maldhari*s’ Livestock Husbandry

We compared the livestock rearing costs by a *Maldhari* herder living within Gir with a livestock herder living outside the forest. *Maldhari* livestock within Gir obtain most of their forage requirements from the forest free of cost, while a major proportion of the fodder for livestock outside the PA needed to be purchased. Occasional predation by lions is the cost of rearing livestock in the Gir forests. We developed a deterministic economic model (Table S2) where we hypothesized that all other costs and profits being equal between the forest dwelling *Maldhari*s and pastoralists living outside, it would be economically profitable for the *Maldhari*s to stay in the forest with lions, if cost of obtaining livestock forage was greater than the economic loss due to lion predation.

The cost of lion predation was estimated in two parts:

- Capital loss- the market price of the predated livestock and
- Lost opportunity cost [72] i.e. the opportunity to earn from the predated livestock in the years to come had it not been killed (Table S2).

Hypothetically this component of cost (b) would occur if there was a deficit between market rates and government compensation paid for different livestock classes predated by lions. We calculated the lost opportunity cost as the amount of income that a *Maldhari* would have made from the predated livestock based on its life expectancy and productivity (Table S2). We modeled two scenarios of *Maldhari*-lion economics; i) with the current state-run predation compensation scheme and ii) without any such compensation scheme to understand the efficacy of the predation compensation scheme in permitting lion-*Maldhari* coexistence inside the Gir forests and its implications for the larger lion-occupied agro-pastoral landscape as well.

## Results

### Lion Density

We obtained 36 sightings of 20 individual lions (3 adult males, 10 adult females and 7 sub-adults). Plot of cumulative number of unique lions against lion sightings reached an asymptote suggesting adequacy of sampling. The model selection procedure of program CAPTURE selected the model incorporating time variation and individual heterogeneity (M_th,_ scored at 1). Program CloseTest supported population closure (χ^2^_12_ = 30.2, P = 0.19). Capture probability of lions was 0.24 and the population estimate under M_th_ was 20±1 SE lions.

Using the ½ MMDM approach, we estimated a buffer width of 2.4±0.2 SE km and an effectively sampled area of 131±17 SE km^2^. Lion density was estimated at 15.2±0.1 SE lions/100 km^2^.

### Livestock Density, Demography and Holding

The average foraging radius of livestock herds of six *ness* sites was 1.9±0.1 SE km. Some foraging areas of two or more *ness*es overlapped i.e. these areas were used by livestock from more than one *ness*es. Therefore, a common buffer of 1.9 km was created on the cluster of *ness* locations to compute livestock density. Livestock foraging area was maximum (95.2 km^2^) in pre-monsoon followed by 76.3 km^2^ during summer and minimum foraging area during winter (65.9 km^2^). All *ness* sites showed seasonal fluctuation in livestock population. Maximum livestock number was observed during monsoon while during winter and summer livestock numbers decreased due to emigration of the herders outside the Gir PA. The livestock density was 31.4/km^2^ in winter, 30.1/km^2^ in monsoon and 24.7/km^2^ during summer.

**Figure 2:**
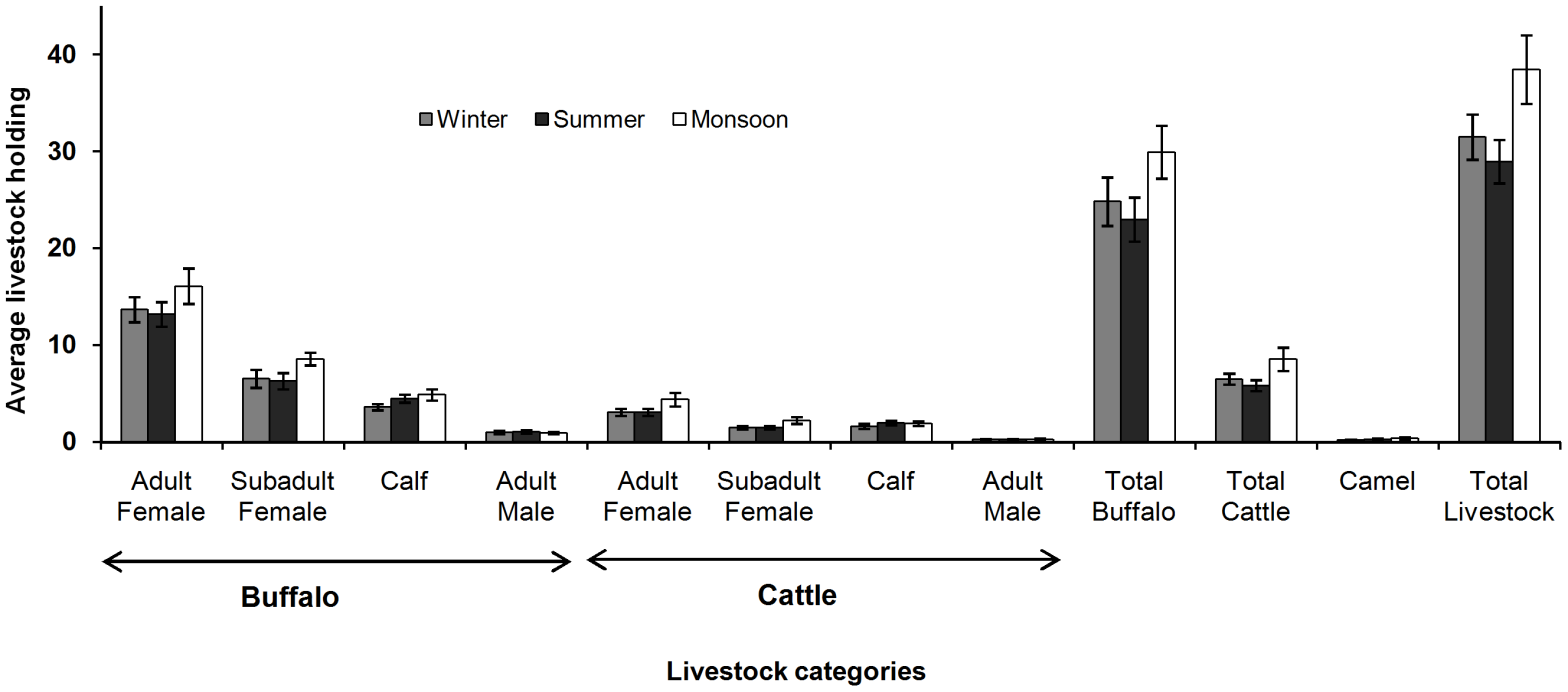
Average seasonal livestock holding of *Maldhari* family within the Gir forests. (Error bars are standard errors).

The total livestock holding of the study *ness*es was 2,140±296 SE. Buffaloes were dominant contributing at 78.1%, while cattle (21.1%) and camels (0.8%) constituted the remainder livestock numbers. Overall population structure of buffaloes and cattle was largely composed of adult and sub-adult females (Fig. 2). Few adult males were kept for breeding purpose. The average livestock holding of a *Maldhari* family varied from 29±3 SE in summer to 31±3 SE in winter and 39±4 SE in the monsoon.

Average grazing herd size was 22±2 SE and was always of mixed composition of cattle and buffaloes. High priced, milk yielding livestock were rarely taken out of the corrals to graze. These were stall fed by forage collected from the forest and by concentrates purchased from the market. Average number of herdsmen accompanying herds was 2±0.04 SE. Spatial lay out of the herds were with cattle (low monetary value) leading, buffaloes (high monetary value) in the middle and juvenile/sub-adult animals (low monetary value) trailing. The herdsmen were usually mobile sometimes leading and at times pushing the herd from the rear.

### Lion Food Habits

**Figure 3:**
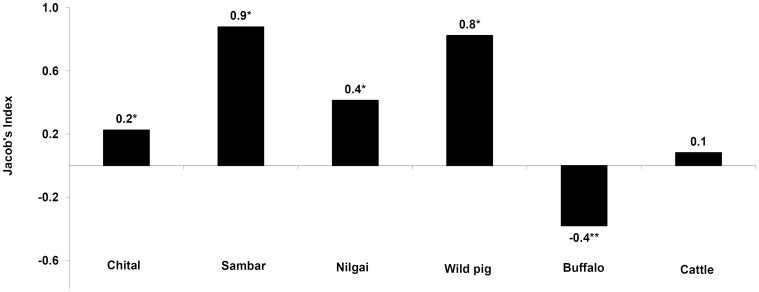
Food preference of lions in the Gir forests, India based on Jacob’s index [59]. Program SCATMAN [55] suggests that at 10% CV * Chital (P<0.001), sambar (P<0.001), nilgai (P<0.05) and wild pig (P<0.001) were found to be positively selected while **buffaloes (P<0.001) were underused in proportion to their availabilities. Cattle (P = 0.33) were utilized in proportion to their availabilities.

Frequency of occurrence of all prey items in scats reached an asymptote after sampling over 130 scats; so our sample size of 165 scats was deemed sufficient. Most (97.6%) lion scat contained a single prey type, while 2.4% of the scats had two prey items. Wild ungulates comprising chital, sambar, nilgai and wild pig together accounted for 76.4% of all prey occurrences, while domestic livestock (buffalo 13.7% and cattle 7.8%) contributed the rest (Table 1). Percentage biomass contribution of different prey species to the lions’ diet was most for livestock (33.7%) followed by chital (28.9%) and sambar (28.3%). There was evidence of selective utilization of prey by lions (G = 76.9, P<0.001, *d.f.* = 5). Chital (χ^2^ = 12.3, P<0.001), sambar (χ^2^ = 103.4, P<0.001), nilgai (χ^2^ = 2.4, P<0.05) and wild pig (χ^2^ = 34.1, P<0.001) were found to be utilized more than their availability while buffaloes (χ^2^ = 60.3, P<0.001) were used less than their availability. Cattle (χ^2^ = 0.9, P = 0.33) were utilized in proportion to their availability. The order of prey preference by lions as estimated by Jacob’s Index was sambar, wild pig, nilgai, chital and cattle (Fig. 3).

**Table 1:** Prey species composition in Asiatic lion *Panthera leo persica* scats (n* = *165) and their relative biomass contribution to lion diet in eastern part of the Gir forests, India.

Prey Items	Body Weight (kg), (x)	Total Number of Scats	Observed Frequency of Occurrence [F] (95% CI)*	Relative Occurrence(as %)	Collectable scats/kill (y)	% Biomass Consumed(95% CI)
Chital	42	72.5	44 (37–51.8)	45	3.5	28.9 (24.3–34.1)
Sambar	119	40	24.4 (17.9–30.6)	24.9	6.2	28.3 (20.9–35.7)
Nilgai	136	7.5	4.6 (1.8–8.5)	4.7	8.3	7.2 (2.8–13.4)
Wild pig	28	5.5	3.4 (1.6–6.8)	3.4	2.9	1.9 (0.8–3.8)
Buffalo	204	22.5	13.7 (8.6–19.1)	13.9	9.1	23.6 (14.8–33)
Cattle	136	13	7.8 (3.7–11.7)	8.1	6.7	10.1 (4.7–14.9)

x and y are related through the equation *y* = 1.98+0.035×[52].

* 95% CIs obtained by 1,000 bootstrapped replicates.

### Livestock Depredation Pattern

**Figure 4:**
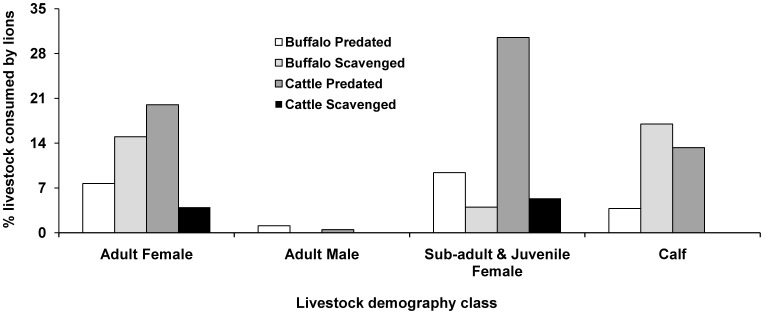
Livestock utilization by lions in the Gir East Sanctuary, India showing percent contribution of different livestock classes in livestock feeding events documented by continuous monitoring on radio-collared lions.

We recorded a total 308 livestock mortalities from the six *ness*es between April 2005 and August 2007, of which 58.4% was due to lion predation, 3.2% was due to predation by leopards and 38.4% was due to other natural causes. Lion predation was mostly on cattle (69.4%) followed by buffaloes (29.4%) and camels (1.2%). Non-productive cattle dominated lion kills (Fig. 4). Average age of livestock predated by lions was estimated at 4±0.2 SE years. Of the 118 events of natural death of livestock, 46.6% were scavenged by lions, mostly adult female buffalo carcasses (27.2%) reflecting a higher availability of this livestock category in the study area.

The 180 lion kills recorded involved 151 successful hunt events [average killed/hunt 1.2±0.5 SE]. The number of livestock killed per successful hunt was weakly correlated with the number of lions reported by the herders (Spearman rank correlation r_s_ = 0.15, P = 0.03). Of the successful lion attack events on livestock, only 13% occurred within the *ness* when lions jumped into the fenced *ness* and killed livestock while 87% occurred in forests when livestock were out grazing. We did not record any leopard attack on grazing herds. In 68 events of lion attacks on grazing herds the herders could affirm the gender of the lions making the kills. Female lions with dependent cubs were responsible for 54.4% of the attacks; single male or male coalitions were responsible for 19.1% of the attacks and mixed groups of lions made 26.4% of the kills. Lionesses in the study area were found to raid livestock in proportion to the prevailing adult sex ratio in the population (χ^2^_1_ = 0.19, P* = *0.66). Thus all lions were equally likely to predate livestock.

Most (49%) of the lion predation events on livestock were recorded during early morning (7 AM –11 AM), followed by 39% in late afternoon (3 PM –7 PM) [χ^2^_2_ = 29.5, P<0.0001]. But during monsoon, most predation events (44%) occurred during late afternoon or evening (χ^2^_2_ = 14.2, P<0.001) due to cooler ambient temperature, poor visibility owing to bad light, rain and thick vegetation undergrowth. Livestock losses to lions were different between seasons (χ^2^_2_ = 6.5, P* = *0.04), with 45% occurring in summer, followed by 30% in monsoon and 25% in winter.

A crude estimate of total intake requirement of the lion population in the study area was about 124,733 kg for the study period of 28 months. Livestock were found to be contributing about 31,582 kg (25.3%) of biomass to the lion’s diet. Inter-feeding interval of lions estimated by the continuous monitoring was 3.5±0.7 SE days with an average associated lion group size during feeding being 4±1 SE. Telemetry data showed that livestock composed 42% of lions’ feeding events (16% from predation and 26% was from scavenging on livestock carcasses). Wild ungulates were found to compose the remainder 58% of lions’ feeding events (47% predated and 11% appropriated from leopard kills or other lion kills).

### Lion Carrying Capacity

Under the assumption of 100% availability of livestock biomass to lion predation, the lion carrying capacity was estimated to be 22 (95% CI 20–25) lions/100 km^2^ while with no availability of livestock, the lion carrying capacity was 12 (95% CI 9–15) lions/100 km^2^. Lion carrying capacity with 24% of livestock population available for lions was 16 (95% CI 13–18) lions/100 km^2^ (Table S3).

### Economics of Lion Predation

Annual fodder cost for maintaining 100 livestock was estimated to be 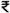
1,460,000 [1US$ ∼ 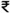
50]. For forest-dwelling *Maldhari* this resource is available free of cost. Average cost of livestock units predated by lion was 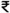
4,018±278 SE. *Maldhari*s incurred an annual capital loss of 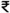
33,751±2,335 SE/100 livestock by lion predation. Sixty four percent of this cost was offset by the government compensation (i.e. capital loss with Government compensation was 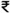
12,150±840 SE/100 livestock). The annual lost opportunity costs incurred by *Maldhari*s was 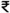
136,156±3,430 SE/100 livestock with Government compensation. The same cost without Government compensation was 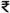
378,212±9,529 SE/100 livestock. By living in the Gir forests, 58±0.2 SE% of livestock rearing cost of *Maldhari*s was accounted for by free forest resources in comparison to a non-forest dwelling pastoralist. With government predation compensation scheme this profit margin was further augmented to 76±0.05 SE% (Table 2). Cost saving (additional profit) by *Maldhari*s living in Gir was therefore, 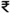
1,104,373/100 livestock/year (or 214 man-day wages/*Maldhari* family/month) and 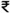
840,717/100 livestock/year (or 163 man-day wages/*Maldhari* family/month) with and without a lion predation compensation scheme respectively in comparison with non-forest dwelling pastoralists (Table S2).

**Table 2:** Parameter values (95% CI) used for the deterministic model of *Maldhari* pastoral economics.

Scenarios	Capital loss/100 livestock/year	Lost Opportunitycost/100 livestock/year	Total revenue loss by lion predation/100 livestock/year	Annual cost savingby living withlions/100 livestock	Percentage benefit as proportion of livestock rearing cost covered by living with lions after accounting for losses due to lion predation
With Government Compensation	12,150 (10,502–13,799)	136,156 (129,432–142,880)	355,626 (353,979–357,275)	1,104,373 (1,102,725–1,106,021)	75.6 (75.5–75.7)
Without Government Compensation	33,751 (29,173–38,329)	378,212 (359,535–396,889)	619,283 (614,705–623,861)	840,717 (836,139–845,295)	57.5 (57.2–57.9)

Final estimates are in Indian Rupees (1 US$ ∼ 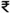
50).

## Discussion

We found that presently *Maldhari* and lions coexist in a win-win state where lions get a considerable part of their food from *Maldhari* livestock and *Maldhari*s profit substantially by free access to forest resources. Average annual financial loss/*Maldhari* household due to livestock predation by lions after offsetting by the compensation was minimal (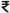
2,038) and was only 5% of the average *per capita* income for Gujarat province and 7% of the national average during the fiscal year 2005–06 [73]. With free grazing rights and at current rate of compensation, additional profits of a *Maldhari* family residing inside Gir approximately amount to a person’s annual minimal wage (213 man-day wages). Current government compensation scheme, though small in comparison to the value of free resources, was important as it provided a *Maldhari* family an additional monthly monetary advantage of 51 man-day wages to a no-compensation scenario (Table S2). We did not, however, consider the additional benefits *Maldhari*s enjoy by dwelling inside Gir i.e. from other ecological services and amenities (collection of fuel wood and minor forest products, use of forest topsoil mixed with dung sold as manure, free access to water, job opportunities with the forest department and maintaining their social customs). These, when incorporated into our analysis, further augment the benefits *Maldhari*s make by living inside Gir.

The *Maldhari*-lion coexistence in Gir forests is long debated with one school of thought attributing ecological deterioration of the Gir to the traditional way of resource usage by *Maldhari*s [74] and therefore advocates their relocation outside the PA. The other school, on the contrary, attributed exclusionary forest policy and insufficient compensation scheme by the Forest Department as causes of economic marginalization of Gir *Maldhari*s [75]. Livestock has always been an important part of lion’s diet in Gir ranging between 83 to 25% [45], [65], [76], [77]. We studied livestock depredation pattern by lions with a combination of methods viz., scat analysis, predation pattern and feeding events of the radio-collared lions in order to address inherent limitations of each method and estimated biomass contribution by domestic livestock in lions’ diet to range between 25 to 42% within eastern Gir PA. Past long-term research from Africa have shown that prey availability and density govern lion demography like cub survival and dispersal rates [63], [78], [79]. Our data suggested that the carrying capacity of lions modeled with available biomass of dead livestock and livestock classes vulnerable to lion predation (24%) was almost similar with the current lion density estimated in the study area (15 lions/100 km^2^). However, when we considered a hypothetical situation where there were no *Maldhari* settlements in the study area and therefore no availability of livestock biomass for lions, the predicted lion carrying capacity went down (12 lions/100 km^2^), albeit not statistically significantly. Moreover, lions in Gir obtained a major part of their diet from scavenging livestock. Being a free resource for lions, this optimized the Gir lions’ energy economics by maximizing the net food intake per unit time available for foraging [80]. Abrupt removal of livestock as a food source is likely to have a detrimental effect on lion density and demography in Gir [37], [81]. We recommend that if removal of livestock is to be considered, it should be in a phased manner so as to allow natural wild prey population to build up and replace livestock [82]. However, diet of wild ungulates in Gir differed substantially from those of livestock [71], [83]; therefore, removal of livestock was unlikely to be fully compensated by increase in wild ungulate biomass. With a lion focused conservation objective of Gir, maintaining livestock at the current or lower stocking densities could also be considered as an alternative management practice. To avoid negative impacts of livestock trampling, livestock numbers should be regulated at the *ness*es with their locations rotated every 4–5 years [42].

Human attitudes towards large carnivores have been shaped by psychology of fear and personal experience [84], and also depend on their attachment to livestock [85]. Gir *Maldhari*s did not view lions as a threat to their lives [11] and there was no lion attack on humans within our study area during past two decades. Moreover, unproductive cattle (such as males and poor condition calves, aged, and dying cattle) were mostly targeted by lion predation. The average cost of such unproductive cattle was 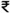
3,425 and at times, it was not profitable to maintain them by stall-feeding. We believe that retaliatory killing of lions is not currently prevalent in Gir due to low economic losses, *Maldhari*s’ cultural ethics, combined with strict legal enforcement by the Gir Park Management. But traditional value systems of the *Maldhari*s are rapidly changing under the influence of globalization and free markets [25], [86]. Younger generations are less tolerant to even small monitory losses which older generations considered as fait-accompli. We anticipate that such changes in attitudes and values are likely to result in a change of *Maldhari*s’ harmonious coexistence with lions. A similar transition has happened with the pastoral Masai community in the eastern Africa within the past two decades [87], [88]. With this change in values, comes complacency towards professional lion poachers by local communities. This was probably the case when 8–10 lions were poached for their body parts in the recent past in Gir [82], [89] and elsewhere in India in the case of tigers, *Panthera tigris* [90], [91]. Reparative measures such as compensation programs become important herein, mitigating conflicts by offsetting monetary costs to local communities [92]. The success of Asiatic lion conservation is partly attributable to the early policies (1930s) of the erstwhile Junagadh Nawabs [93] and later to the state run Gujarat Forest Department in implementing compensation schemes for livestock predation [82]. In order to reflect the current market value of the livestock, the compensation rate is usually revised at an interval of every 6–8 years [82]. We found that the current compensation scheme substantially minimized lion-*Maldhari* conflicts by lowering the latter’s capital loss by 64% and allowing them to make an additional monthly monetary profit of 51 man-day wages/family in comparison to a non-compensation scenario. We believe that this had a positive role in shaping *Maldhari*s’ perceptions about their personal losses and thus acts as an important factor promoting their coexistence with lions. A similar pattern has been observed among the Masai community residing around the Mbirkani Ranch, Kenya where individuals receiving compensation from a local NGO showed a lower propensity to kill lions and were found to bear more positive attitude towards conservation [94], [95]. The current compensation scheme in Gir addresses *Maldhari*s’ capital loss to a significant extent. Increasing this to current market value of the predated livestock by timely revision (every 2 years) would ensure that there is no lost opportunity cost to the local communities. However, recognizing the role of compensation policies in providing instant financial relief, the procedural framework of the current system in Gir could be more streamlined and provisions of onsite payments with active involvement of local non-governmental organizations like that prevailing in Corbett and Dudwa Tiger Reserves, India [96] could also be adopted.

*Maldhari*s and Masai seemed to have mastered husbandry practices over generations to minimize predation losses to lions and permit coexistence. Both communities corral their livestock at night in their ‘bomas’ and graze the livestock during daytime, avoiding peak lion activity period and having expert herdsmen [97]. In Gir, cattle were the preferred prey of lions as they are easy to kill due to their behavior of flight when attacked while buffaloes have a defense strategy and often attack lions as a cohesive group [98]. Cattle are relatively less priced in comparison to buffaloes and therefore *Maldhari* grazing herds were always observed to have a few non-productive cattle. Thus, when lions attack, they are more likely to kill these vulnerable cattle. Moreover, *Maldhari* herdsmen orient their herds with cattle leading, buffaloes in the middle and juvenile animals trailing. We speculate that the current traditional mechanism of warding off lion predation by corralling livestock at night and having a mixed grazing herd composition being always accompanied by expert herdsmen minimized the risks and economic losses to lion predation. In Gir since livestock are reared only for dairy products and are not consumed by *Maldhari*s [11], [33] there is a large cohort of old and weak cattle in which natural mortality is high and these carcasses are available to lions for scavenging.

We conclude that the underlying economics of *Maldhari* livelihood securities, their religious sentiments, ecological benefits enjoyed by pastoralists living in lion habitats and strict legal protection regime for lions in the Gir forests [11], [31], [70] are all needed as recipe for lion-*Maldhari* coexistence. Indefinite increase in human and livestock population within the Gir forests would upset this balance by altering the forest composition or even population dynamics of wild prey [99] and would thus be detrimental for the conservation objective of the Protected Area. Presently lions are dispersing out of the Gir PA and have already occupied about 9,000 km^2^ of agro-pastoral landscape [28], [29], [41]. Our ongoing telemetry study suggests that lions outside the PA depend substantially on livestock, thereby increasing the chances of human-lion conflict in the region [81], [100]. In the agro-pastoral landscapes, there are no free economic benefits for the communities. Government compensation scheme therefore becomes extremely crucial for maintaining the goodwill of the communities towards lion conservation.

Due to high human densities and demand for land most human free inviolate protected areas in India and elsewhere are too small to hold viable populations of large carnivores for the long-term [101], [102]. Coexistence with humans therefore becomes essential if large carnivores were to be conserved for the long-term. Considering the case of Asiatic lions, only about 10% of the lion population resides in the human-free Gir National Park, 62% of lion population resides in the Gir Sanctuary (with *Maldhari* settlements) while 22% of the adult lion population resides in the human-dominated agro-pastoral landscape of Saurashtra [81], [103]. A comparable situation exists with many tiger populations in India as well [104]. Such scenarios are common to several developing countries and activities like paying compensation should be considered as ecosystem maintenance costs that need to be paid to the local communities by Global societies or Governments for the continued survival of large carnivores within landscapes of conflict to promote coexistence. This would foster greater tolerance by local communities towards lion conservation in the Gir landscape and for other large carnivores elsewhere. We see no end to this or similar programs worldwide and believe that they form an integral component of coexistence and an important component of conserving viable populations of large carnivores.

## Supporting Information

Table S1
**Average monetary values (Indian Rupees, 1 US$ ∼ 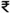
50 ) for various age-sex-productivity categories of livestock (buffalo and cattle) used for analysis.** The values in parentheses show the compensations amounts paid by the state forest department for the respective livestock classes to offset economic loss due to predation by carnivores in and around the Gir Protected Area (after [71]).(PDF)

Table S2
**Description and estimation of cost parameters used for economic analysis. Final estimates are in Indian Rupees** (1 US$ ∼ 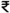
50).(PDF)

Table S3 **Predicted carrying capacity of Asiatic lions in the eastern part of the Gir forests at different availability of livestock biomass.** Lion carrying capacity was predicted using the equation y = −2.158+0.377×(r^2^ = 0.71, n = 23) where y is the log_10_ of lion density and *x* is the log_10_ of prey biomass [57]. Figures within parentheses are 95% CIs. Densities of wild ungulates (chital, sambar, nilgai and wild pig) were taken from literature [38].
(DOC)
