# Correction: Living with Lions: The Economics of Coexistence in the Gir Forests, India

**DOI:** 10.1371/journal.pone.0089708

**Published:** 2014-02-26

**Authors:** The PLOS ONE Staff

This article was republished on January 27, 2014, due to missing symbols in the text. Please download the article again to view the correct version. The originally published, uncorrected article is provided here as File S1 for reference.

## Supporting Information

File S1 Originally published, uncorrected article.
(PDF)
