# Supplementary material for: Living with Lions: The Economics of Coexistence in the Gir Forests, India
Source: PLoS One. 2013 Jan 16;8(1):e49457. doi: 10.1371/journal.pone.0049457 (PMC3547023; doi:10.1371/journal.pone.0049457)
Supplement: Table S1 — Average monetary values (Indian Rupees, 1 US$ ∼ 50 ) for various age-sex-productivity categories of livestock (buffalo and cattle) used for analysis. The values in parentheses show the compensations amounts paid by the state forest department for the respective livestock classes to offset economic loss due to predation by carnivores in and around the Gir Protected Area (after [71]). (PDF) [file pone.0049457.s001.pdf]

Table S1 Average monetary values (Indian Rupees ₹, 1 US$ ~ ₹ 50) for various age-sex-productivity categories of livestock (buffalo and cattle) used for analysis. The values in parentheses show the compensations amounts paid by the state forest department for the respective livestock classes to offset economic loss due to predation by carnivores in and around the Gir Protected Area (after [82])

	Male calf	Female calf	Juvenile male	Juvenile female	Sub-adult male	Sub-adult female	Milk yielding adult female	Dry productive adult female	Non-productive adult female	Adult male	Non-productive adult male
Buffalo	800 (1,100)	1,000 (1,500)	1,200 (1,100)	2,500 (1,500)	3,500 (3,500)	4,000 (2,100)	15,000 (8,000)	10,000 (2,100)	4,000 (1,100)	5,000 (3,500)	3,000 (1,100)
Cattle	500 (1,100)	800 (1,100)	500 (1,100)	1,200 (1,100)	2,000 (3,500)	3,500 (2,100)	8,000 (6,000)	5,000 (1,100)	1000 (1,100)	8,000 (6,000)	500 (1,100)
