# Supplementary material for: Living with Lions: The Economics of Coexistence in the Gir Forests, India
Source: PLoS One. 2013 Jan 16;8(1):e49457. doi: 10.1371/journal.pone.0049457 (PMC3547023; doi:10.1371/journal.pone.0049457)
Supplement: Table S2 — Description and estimation of cost parameters used for economic analysis. Final estimates are in Indian Rupees (1 US$ ∼ 50). (PDF) [file pone.0049457.s002.pdf]

Table S2 Description and estimation of cost parameters used for economic analysis. Final estimates are in Indian Rupees ₹ (1 US$ ~ ₹ 50). Values within parentheses are 95% CIs.

Equation Number	Parameters	Parameter Description	Final Estimates (95% CI)
A	Fodder cost for 100 livestock (LS)/year	8 kg fodder/LS/day @ ₹ 5/kg for 365 days for 100 livestock	1,460,000
B	Loss of capital due to lion predation (with Govt. compensation) per 100 LS/year	(Average cost of livestock predated – Government compensation received) X (% of LS killed by lions)	12,150 (10,502 – 13,799)
C	Loss of capital due to lion predation (without Govt. compensation) per 100 LS/year	(Average cost of livestock predated) X (% of LS killed by lions)	33,751 (29,173 - 38,329)
D	Lost opportunity cost due to lion predation with Govt. compensation	Computed by considering the proportion of productive LS (0.28) in the population and the average potential life of a predated livestock and its productivity within that span. The total average life expectancy i.e. 12 yr – average age of predation i.e. 3.9 yrs) was used to compute life remaining. Predation rate of lions on 100 livestock was used to compute numbers killed. These were corrected for by percent of natural mortality (i.e.	136,156 (129,432 – 142,880)

		lion predation of 8.4%, while natural mortality of 5.5% of 8.4% was 0.4%). Annual profit/LS from milk was computed as 200 days of milk yield annually of an average yield of 5 liters/LS and a cost of ₹ 20/liter. A livestock was considered to calve once each year with and equal sex ratio amongst calves. An average calf was computed to cost ₹ 845 (considering the proportion of cattle and buffaloes in the LS population) and this was added to the milk production i.e. ((5*20*200) + 845). With government compensation scheme for livestock predation 64% of this cost gets compensated and remaining 36% was uncompensated. Therefore the final equation becomes (12-3.9)*(8.4-0.4)*[(5x20x200) + 845]* 0.28*0.36	
E	Lost Opportunity cost without compensation	Same as equation D but with 100% of the cost being uncompensated. Therefore the final equation becomes (12-3.9)*(8.4-0.4)*[(5x20x200) + 845]* 0.28	378,212 (359,535 – 396,889)

F	Guarding cost for *Maldhari* (Assuming that outside herder will need half the herders than a *Maldhari* in lion habitat)	1 person extra for a herd of 25 therefore for 100 LS the cost is of 4 persons@ ₹ 142[§] /day for a year	207,320
G	Total revenue loss to *Maldhari* by lion predation & extra guard cost when Govt. compensation is availed for lion predation	B+D+F	355,626 (353,979 – 357,275)
H	Total revenue loss to *Maldhari* by lion predation & extra guard cost when Govt. compensation is not availed for lion predation	C+E+F	619,283 (614,705 – 623,861)
I	Profit of living in lion habitat with Govt. compensation for lion predation	A-G	1,104,373 (1,102,725 – 1,106,021)
J	Profit of living in lion habitat without Govt. compensation for lion predation	A-H	840,717 (836,139 – 845,295)
K	% of cost offset by living in Gir forest with Govt. compensation	100*(I/A)	75.6 (75.5 – 75.7)

L	% of cost offset without compensation	100*(J/A)	57.5 (57.2 – 57.9)
Average livestock holding in a *Maldhari* family			33
M	Total monthly cost for rearing 33 LS units outside Gir would be	[(A/100)*33]/12	40,150
N*	By living in the Gir forest this is reduced by 58%, therefore the monthly monetary gain	M-(1-L)*M	162.8 (161.9 – 163.7)
O*	By lion predation Govt. compensation the monthly rearing cost is reduced by 82%, therefore monthly actual monetary advantage	M-(1-K)*M	213.8 (213.5 – 214.2)
	Additional monetary advantage due to compensation*	N-O	51

[§] Daily labor's wage rate of the Gujarat State Government Labor Department during the study period.

*Final estimates are in man-day units which were calculated by dividing the monthly monetary gains in ₹ by labor rate i.e. ₹ 142.
