# Supplementary material for: Living with Lions: The Economics of Coexistence in the Gir Forests, India
Source: PLoS One. 2013 Jan 16;8(1):e49457. doi: 10.1371/journal.pone.0049457 (PMC3547023; doi:10.1371/journal.pone.0049457)
Supplement: Table S3 — Predicted carrying capacity of Asiatic lions in the eastern part of the Gir forests at different availability of livestock biomass. Lion carrying capacity was predicted using the equation y = −2.158+0.377×(r2 = 0.71, n = 23) where y is the log10 of lion density and x is the log10 of prey biomass [57]. Figures within parentheses are 95% CIs. Densities of wild ungulates (chital, sambar, nilgai and wild pig) were taken from literature [38]. (DOC) [file pone.0049457.s003.doc]

Table S3 Predicted carrying capacity of Asiatic lions in the eastern part of the Gir forests at different availability of livestock biomass. Lion carrying capacity was predicted using the equation y = -2.158 + 0.377x (r^2^ = 0.71, n = 23) where y is the log_10_ of lion density and x is the log_10_ of prey biomass [66]. Figures within parentheses are 95% CIs. Densities of wild ungulates (chital, sambar, nilgai and wild pig) were taken from literature [42].

| Prey species | Density/km^2^ | Body weight (kg) | 100% livestock biomass available | | | No livestock biomass available | | | 24% livestock biomass available | | |
| --- | --- | --- | --- | --- | --- | --- | --- | --- | --- | --- | --- |
|  |  |  | Biomass density/km^2^ | Log (prey biomass) | Predicted lion carrying capacity (lions/100km^2^) | Biomass density/km^2^ | Log (prey biomass) | Predicted lion carrying capacity (lions/100km^2^) | Biomass density/km^2^ | Log (prey biomass) | Predicted lion carrying capacity (lions/100km^2^) |
| Chital | 35.1  (16.0 - 54.5) | 42 | 1473.4  (673.7 – 2,289) | 3.2  (2.8 – 3.4) | 22  (20 – 25) | 1473.4  (673.7 – 2,289) | 3.2  (2.8 – 3.4) | 12  (9 – 15) | 1473.4  (673.7 – 2,289) | 3.2  (2.8 – 3.4) | 16  (13 – 18) |
| Sambar | 2.9  (1.3 – 4.4) | 119 | 340.3  (153.5 – 527.2) | 2.5  (2.2 – 2.7) |  | 340.3  (153.5 – 527.2) | 2.5  (2.2 – 2.7) |  | 340.3  (153.5 – 527.2) | 2.5  (2.2 – 2.7) |  |
| Nilgai | 1.2  (0 – 3.4) | 136 | 159.1  (0 – 455.7) | 2.2  (0 – 2.7) |  | 159.1  (0 – 455.7) | 2.2  (0 – 2.7) |  | 159.1  (0 – 455.7) | 2.2  (0 – 2.7) |  |
| Wild pig | 0.4  (0 – 1) | 28 | 11.2  (0 – 27.7) | 1.0  (0 – 1.4) |  | 11.2  (0 – 27.7) | 1.0  (0 – 1.4) |  | 11.2  (0 – 27.7) | 1.0  (0 – 1.4) |  |
| Buffalo | 32.8  (26.6 – 39.1) | 204 | 6,699.4  (5,420.3 – 7,978.4) | 3.8  (3.7 – 3.9) |  | -- | -- |  | 1,599.8  (1,294.4 – 1,905.3) | 3.2  (3.1 – 3.3) |  |
| Cattle | 9.2  (7.5 – 11) | 136 | 1,256.4  (1,017.3 – 1,496) | 3.1  (3.0 – 3.2) |  | -- | -- |  | 300.1  (242.9 – 357.2) | 2.5  (2.4 – 2.6) |  |
